# Understanding burnout according to individual differences: ongoing explanatory power evaluation of two models for measuring burnout types

**DOI:** 10.1186/1471-2458-12-922

**Published:** 2012-10-30

**Authors:** Jesús Montero-Marín, Ricardo Araya, Barbara Olivan Blazquez, Petros Skapinakis, Vicente Martinez Vizcaino, Javier García-Campayo

**Affiliations:** 1 Faculty of Health Sciences and Sports, University of Zaragoza, Huesca, Spain; 2 School of Community and Social Medicine, University of Bristol, Bristol, United Kingdom; 3 Faculty of Social and Labour, University of Zaragoza, Zaragoza, Spain; 4 Department of Psychiatry, University of Ioannina School of Medicine, Ioannina, Greece; 5 Health and Social Research Center, Castilla-La Mancha University, Cuenca, Spain; 6 Department of Psychiatry, University of Zaragoza, Zaragoza, Spain; 7 REDIAPP “Red de Investigación en Actividades Preventivas y Promoción de la Salud”, (Research Network on Preventative Activities and Health Promotion) (RD06/0018/0017), Avda Gomez Laguna 52, 4D, Zaragoza, 50.009, Spain

**Keywords:** Burnout, Subtypes, Explanatory power, BCSQ-36, BCSQ-12, MBI-GS

## Abstract

**Background:**

The classic determination of burnout is by means of the dimensions exhaustion, cynicism and inefficacy. A new definition of the syndrome is based on clinical subtypes, consisting of “frenetic” (involved, ambitious, overloaded), “underchallenged” (indifferent, bored, with lack of personal development) and “worn-out” (neglectful, unacknowledged, with little control). The dimensions of overload, lack of development and neglect form a shortened version of this perspective. The aims of this study were to estimate and to compare the explanatory power of both typological models, short and long, with the standard measurement.

**Methods:**

This was a cross-sectional survey with a randomly sample of university employees (n=409). Multivariate linear regression models were constructed between the “Maslach Burnout Inventory General Survey” (MBI-GS) dimensions, as dependent variables, and the “Burnout Clinical Subtype Questionnaire” (BCSQ-36 and BCSQ-12) dimensions, as independent variables.

**Results:**

The BCSQ-36 subscales together explained 53% of ‘exhaustion’ (p<0.001), 59% of ‘cynicism’ (p<0.001) and 37% of ‘efficacy’ (p<0.001), while BCSQ-12 subscales explained 44% of ‘exhaustion’ (p<0.001), 44% of ‘cynicism’ (p<0.001), and 30% of ‘efficacy’ (p<0.001). The difference in the explanatory power of both models was significant for ‘exhaustion’ (p<0.001), and for ‘cynicism’ (p<0.001) and ‘efficacy (p<0.001).

**Conclusions:**

Both BCSQ-36 and BCSQ-12 demonstrate great explanatory power over the standard MBI-GS, while offering a useful characterization of the syndrome for the evaluation and design of interventions tailored to the characteristics of each individual. The BCSQ-36 may be very useful in mental health services, given that it provides a good deal of information, while the BCSQ-12 could be used as a screening measure in primary care consultations owing to its simplicity and functional nature.

## Background

Rationalization in production systems has led to significant transformations in the structure of the economic sector in modern societies. New features of the labour market, such as the more unstable nature of recruitment and work contracts, may have contributed to the increased vulnerability of workers to stress, with serious consequences to their health [1]. In fact, between 50% and 60% of sick leave taken in the European Union (EU) is caused by work-related stress, and the economic cost to the EU amounts to about 20 billion euros [2]. Currently, one of the most significant work-related problems resulting from stress is burnout syndrome, which causes considerable social and economic losses [3].

Burnout poses a psychosocial risk with negative consequences both for subjects suffering from it and the organizations for which they work. It can affect an individual’s physical and/or mental health, giving rise to psychosomatic disorders such as cardio-respiratory alterations evere headaches, gastritis, ulcers, insomia, dizziness, etc., or psychopathological disorders such as anxiety, obsession-compulsion, interpersonal sensitivity, depression, hostility, paranoid ideation, alcoholism and addictions. For organizations, it can lead to serious reduction in performance and productivity, deterioration in customer service, excessive rotations and absenteeism, and can even lead to employees leaving their jobs [4].

According to the most widely-used definition proposed by Maslach, Schaufeli and Leiter [5], this syndrome includes the dimensions of exhaustion, cynicism and professional inefficacy, and is the result of prolonged exposure to chronic stressors in the workplace. According to this model, ‘exhaustion’ is the feeling of not being able to offer any more of oneself at an emotional level; ‘cynicism’ represents a distant attitude towards work, those served by it, and other colleagues; and ‘inefficacy’ as the feeling of not performing tasks adequately or being incompetent at work [5,6]. This emphasis on aspects of the individual symptoms imposed by the classic approach has not encouraged the development of intervention programmes with positive long and medium-term results on individuals and organizations overall [7-9]. Later approaches based on this classic model have allowed distinctions to be made with respect to how this syndrome presents depending on the presence or absence of psychological symptoms, such as guilt [10], which has opened up the possibility of dealing with the disorder from the perspective of individual differences.

Nevertheless, when studying burnout syndrome, and in order to achieve an comprehensive understanding of this phenomenon, we should take into consideration the way in which subjects are affected by environmental stressors [11]. In other words, we need to assess the characteristics of both individuals and their environment, given that burnout should be understood not as a purely intrapsychic phemomenon, but as the result of a social practice, in a cultural, economic and political context [12]. In this regard, a definition of the syndrome that is well integrated on a psychosocial level, such as that provided by social exchange theory, could facilitate the design of holistic intervention programmes to a greater degree. According to the social exchange theory, burnout seems to be strongly influenced by a perception of lack of reciprocity in social exchange relationships [13]. The perception of an imbalance between efforts-rewards is an important source of stress at work [14] and can be seen as a determinant risk factor for the development of burnout symptoms [15,16].

Clinical experience suggests different ways for the disorder to become manifest. As Faber proposed, from a phenomenological orientation framed within the viewpoint of the exchange [17-23], burnout has been described as an experience where workers sense a strong feeling of discontentment owing to the discrepancy between their personal contributions and the gratification obtained in return. The level of dedication given to the job-related tasks that provoke such feelings of frustration could determine the development of different burnout subtypes. Consequently, the degree of dedication to job-related tasks forms a classification criterion that is able to integrate a new conceptual framework for the development of burnout by means of subtypes: “frenetic” (high dedication or active coping style), “underchallenged” (intermediate dedication) and “worn-out” (low dedication or passive coping style) [23-26]. This new perspective of the specific development course of the syndrome, has been put forward as the result of a detailed clinical observations and systemized by means of analysis of the qualitative content. It has also been operationally defined in quantitative terms through the “Burnout Clinical Subtype Questionnaire” or BCSQ-36. The validity of the content of this instrument is based on the experiences reported by patients affected by the syndrome. The factorial structure and internal consistency of the BCSQ-36 has been tested with good results [25]. This new model has also been presented in a short form as the BCSQ-12, with satisfactory results related to construct validity and predictive capacity in samples of both workers [27] and students [28]. Table 1 gives the dimensions of burnout in the classic or standard perspective provided by the “Maslach Burnout Inventory General Survey” (MBI-GS) as well as the properties of the typological model, as seen in the BCSQ-36 and BCSQ-12.

**Table 1:** Burnout according to the MBI-GS, BCSQ-36 and BCSQ-12 models

**MBI-GS**	**BCSQ-36**	**BCSQ-12**
Exhaustion	Frenetic	Overload
	*Overload*	
	*Ambition*	
	*Involvement*	
Cynicism	Underchallenged	L. Development
	*L. Development*	
	*Indifference*	
	*Boredom*	
Inefficacy	Worn-out	Neglect
	*Neglect*	
	*L. Acknowledgement*	
	*L. Control*	
		

*MBI-GS* Maslach Burnout Inventory General Survey, *BCSQ-36* Burnout Clinical Subtype Questionnaire in its long version, *BCSQ-12* Burnout Clinical Sutype Questionnaire in its short version.

Subjects classified as the “frenetic” subtype work increasingly harder, to the point of exhaustion, in search of success. These are highly involved, ambitious and overloaded workers, who invest a great deal of time in their work. ‘Involvement’ is the investment of every effort required to overcome difficulties; ‘ambition’ is a great need to obtain important success and achievements at work; and ‘overload’ is risking one’s own health and neglecting of one’s own personal life in the pursuit of good results. The "frenetic" burnout subtype feels stressed as a result of overload, mainly owing to the subject’s exessive involvement and ambition. These characterstics act as significant predictors of burnout in the classic sense of the word, given that in order to burn out, one would have to “be set alight” [4,17,21,24-26,29]. Workers developing the “underchallenged” burnout subtype have to cope with monotonous and unstimulating conditions that fail to provide the necessary satisfaction. They feel limited by their type of work, and feel indifferent and bored; feelings which do not encourage personal development in their jobs. ‘Indifference’ is lack of interest and enthusiasm in work-related tasks; ‘boredom’ is caused by the understanding of work as a mechanical and routine experience with little variation in activities; and ‘lack of development’ is the absence of personal growth experiences for individuals together with their desire for taking on other jobs where they can better develop their skills. The “underchallenged” burnout subtype has lost all enthusiasm for work, leading the subject to carry out tasks with indifference. This is the result of stress caused by boredom and the lack of personal development, properties that are important antecedents of the syndrome and which are seen as a particular form of antecedent [17,21,24-26,29,30]. Workers presenting the “worn-out” subtype give up when faced with stress or absence of gratification. They are negatively influenced by the effect accumulated over time related to the rigidity of the organizational structure of their employing institution, and show feelings of lack of control. They feel there is lack of acknowledgement of their efforts, leading them to neglect their responsibilities. ‘Lack of control’ is the feeling of helplessness as a result of dealing with many situations that are beyond their control; ‘lack of acknowledgement’ is the belief that the organizations those individuals work for fail to take their efforts and dedication into account; and ‘neglect’ refers to individuals’ disregard as a response to any difficulty. The “worn-out” subtype deals with work-related responsibilities with certain neglect, as a way in which the subject passively copes with the stress of experiencing lack of control in his/her work and the absence of acknowledgement for efforts, experiences that have been described as important predictors for the syndrome [16,17,21,24-26,29]. In general terms, the characteristics of the subtypes are modifiable properties that contribute to the differential development of the disorder and provide us with an idea of how the environmental conditions of the workplace contribute to the development of the syndrome when affecting as stressors.

This typological approach contrasts with the traditional definition, which is more orientated towards offering an unitary (albeit three-dimensional) definition of the syndrome, with more or less consistent aetiology and symptoms [5,6]. In turn, the typological approach is distinguished by the possibility it offers when it comes to identifying the different ways on which the disorder is manifested, enabling their evaluation and the development of interventions adjusted to the particular medical history of each case [25-28]. The rationale for this is not to differentiate between clinical and non-clinical cases of burnout; rather, this approach provides information related to the relevant characteristics from a clinical perspective, seeing as they have been referred to spontaneously during the course of therapy as sources of psychological distress [17-24].

The “frenetic” profile is associated with the classic dimension of ‘exhaustion’, which is understandable if we consider the excessive workload experienced by this highly dedicated type of subject [4,18,31-34], for which it was established that the dimensions that characterize this profile (‘involvement’, ‘ambition’ and ‘overload’) could contribute to a greater extent than the others in the explanation of ‘exhaustion’. The “underchallenged” profile is related to the classic dimension of ‘cynicism’, possibly owing to the subject’s lack of enthusiasm resulting from their negative appraisal of their work conditions [30,31,35-37], for which it was established that the dimensions characterizing this profile (‘indifference', ‘boredom’ and ‘lack of development’) could contribute to a greater extent than the others in explaining ‘cynicism’. The “worn-out” profile is associated with the classic dimension of ‘inefficacy’, probably owing to subjects’ apathy and lack of commitment [25,31,38,39], accentuated by experiences of lack of control and lack of acknowledgement [40-43], for which it was established that the dimensions that characterize it (‘neglect’, ‘lack of control’ and ‘lack of acknowledgement’) could contribute to a greater extent that the others in explaining ‘inefficacy’. Finally, owing to the fact that the long BCSQ-36 model includes a larger number of factors than the short BCSQ-12 (only consisting of the dimensions of ‘overload’, lack of development’ and ‘neglect’), it was established that the explanatory power of BCSQ-36 could be greater than that of BCSQ-12 in relation to the standard dimensions of 'exhaustion', ‘cynicism’ and ‘inefficacy'.

The aim of this work was to estimate and compare the explanatory power of the new typological perspective of burnout through its long and short versions with the standard model, assessing the individual contribution from the properties making up both typological models. Shedding light on these points may contribute to the establishment of their possible differential usefulness, providing understanding of the process by which the syndrome develops by means of the different burnout profiles.

## Methods

### Study design

We used a cross-sectional design to conduct an online self-assessment survey completed by participants who had previously given their informed consent.

### Participants

The study population consisted of all employees of the University of Zaragoza working in January 2008 (N=5,493), in order to make up a multi-occupational group in jobs of a very different nature. These workers form a population at risk from developing burnout, as they consist of professionals working face to face with other people [4]. The required sample size was calculated so as to be able to make estimates with a 95% confidence level, 3.5% margin for error and assuming an 18% prevalence of burnout [44], resulting in a need of sample of 427 subjects. On previous web-mail surveys the response rate was roughly 27% [45,46]. Therefore, 1,600 subjects were selected by means of random stratified sampling with proportional allocation depending on occupation (58% teaching and research staff, from now on referred to as ‘TRS’, 33% administration and service personnel, from now on referred to as ‘ASP’, and 9% grant holders, from now on referred as ‘GRH’) from an alphabetical list of the entire workforce. Sample size calculation and random sampling were performed with Epidat 3.1 software.

### Procedure

In February 2008 an e-mail was sent to the selected subjects explaining the aims of the research. This message contained a link to the online questionnaire and access passwords for subjects to complete the questionnaire. All participants received an anonymous report with an explanation of their results. The project was approved by the regional Clinical Research Ethics Committee of Aragon.

### Measurements

#### Sociodemographic and occupational characteristics

Subjects were first asked to complete a series of specifically-prepared questions related to general sociodemographic and occupational characteristics. The questionnaire collected information on the variables: age, gender, stable relationship (‘yes’ vs. ‘no’), children (‘yes’ vs. ‘no’), level of education (‘secondary or lower’, ‘university’, ‘doctorate’), occupation (‘TRS’, ‘ASP’, ‘GRH’), number of hours worked per week (‘< 35’, ‘35-40’, ‘> 40’), length of service (‘< 4 years’, ‘4-16 years’, ‘> 16 years’), monthly income (‘< €1,200’, ‘€1,200-2,000’, ‘> €2,000’), sick leave taken in the previous year (‘yes’ vs. ‘no’), contract duration (‘permanent’ vs. ‘temporary’) and contract type (‘full time’ vs. ‘part time’).

#### Standard burnout

Subjects were presented with the MBI-GS [6] in its validated Spanish language version [47]. This adaptation consists of 15 items grouped into three dimensions: ‘exhaustion’,‘cynicism’ and ‘efficacy’. Responses were arranged in a Likert-type scale with 7 response options, scored from 0 (‘never’) to 6 (‘always’). The ‘exhaustion’ dimension consists of 5 items (e.g. “I feel emotionally drained from my work”), the ‘cynicism’ dimension consists of 4 items (e.g. “I've become more callous toward people since I took this job”) and the ‘efficacy’ dimension consists of 5 items (e.g. “I deal very effectively with the problems of my work”). The results of each of the dimensions were presented as scaled scores. Both the factorial validity of the MBI-GS and internal consistency of the dimensions comprising it were adequate [47].

#### Burnout subtypes

They were then asked to complete the BCSQ-36 [19] in its Spanish language version [21]. This questionnaire consists of 36 items evenly distributed into 3 scales and 9 subscales (comprising 4 items in each). The “frenetic” scale assessed the ‘involvement’ (e.g. “I react to difficulties in my work with greater participation”), ‘ambition’ (e.g. “I have a strong need for important achievements in my work”) and ‘overload’ (e.g. “I overlook my own needs in order to fulfil work demands”) dimensions; the “underchallenged” scale consisted of the ‘indifference’ (e.g. “I feel indifferent about my work and have little desire to succeed”),‘lack of development’ (e.g. “My work doesn’t offer me opportunities to develop my abilities”) and ‘boredom’ (e.g. “I feel bored at work”) dimensions; and the “worn-out” scale enquired about the ‘neglect’ (e.g. “When things at work don’t turn out as well as they should, I stop trying”), ‘lack of acknowledgement’ (e.g. “I think my dedication to my work is not acknowledged”) and ‘lack of control’ (e.g. “I feel the results of my work are beyond my control”) dimensions. This questionnaire also includes the short version, BCSQ-12 [22], made up of 12 items consisting solely of the dimensions ‘overload’, ‘lack of development’ and ‘neglect’. Subjects had to indicate the degree of agreement with each one of the statements presented according to a Likert-type scale with 7 response options, scored from 1 (‘totally disagree’) to 7 (‘totally agree’). The scores from each of the dimensions were presented as a sum of its constituent items divided by the number of items (scaled score). The factorial validity of the BCSQ-36 and BCSQ-12 presented consistent results, with α≥0.80 reliability in each of their constituent dimensions [19,22].

### Data analysis

A descriptive analysis was made of participants’ sociodemographic and occupational characteristics, using means and percentages according to the nature of the variables.

The explanatory power of the the BCSQ-36 and BCSQ-12 in relation to the standard MBI-GS was assessed by means of the construction of multiple linear regression models. For this purpose, the MBI-GS subscales ‘exhaustion’, ‘cynicism’ and ‘efficacy’ were considered variable dependents, while the BCSQ-36 subscales of ‘involvement’, ‘ambition’, ‘overload’, ‘indifference’, ‘lack of development’, ‘boredom’, ‘neglect’, ‘lack of acknowledgement’ and ‘lack of control’ and the BCSQ-12 subscales of ‘overload’, ‘lack of development’ and ‘neglect’ were considered independent variables. Six models in total were consequently constructed.

The predictive capacity of those models was examined taking into account standard errors (Se) and evaluating goodness of fit by means of analysis of variance associated with the regression analysis, through the calculation of the significance of the F value (df_1_/df_2_). Multiple correlation coefficients (R_y.123_) were calculated to quantify the degree of association between each dependent variable and the independent variables taken as a set. Multiple determination coefficients (R^2^_y.123_) and adjusted multiple determination coefficients (adj-R^2^_y.123_) were also calculated to evaluate and compare the explanatory capacity of the BCSQ-36 and BCSQ-12. Greater confidence was given to the adj-R^2^_y.123_ coefficient as it was the best estimator for the percentage of explained variance, and given that this coefficient takes into account the number of variables included in the equation, which enabled the incidence of accumulated random effects to be counteracted, making this particularly adequate when it came to comparing the predictive capacity of different models [48,49]. The result of those comparisons was assessed, with estimation of the significance of the F value associated with the increase in the adjusted multiple coefficient of determination (Δ-adj-R^2^_y.123_), when going from the short model provided by BCSQ-12 to the long model proposed by BCSQ-36. The ‘raw’ relationship of each independent variable with each dependent variable was calculated by applying Pearson’s r correlation coefficient. The correlations between all the subscales were generally shown through the calculation of this coefficient. The individual contribution of the independent variables in each multivariate model was estimated by means of the calculation of slopes (B), their standard errors and 95% confidence interval (95% CI), and of the standardized slope coefficents (Beta). Partial correlation coefficients (R_y3.12_) were also calculated, indicating the correlation between two variables when the effect of the other variables included in the equation is removed. Semi-partial correlation coefficients (R_y(3.12)_) were also calculated, the square of which shows the increase in the coefficient of determination after including a specific variable in a model, partializing the influence of the other included variables. The Wald test was used to evaluate the statistical significance of the contribution of each variable to each multivariate model.

Tolerance (T) values were calculated in order to rule out possible collinearity errors. These means represent the percentage of each variable that is not explained by the remaining variables; high scores suggest that the variables are independent and help avoid mistaken estimations in the coefficients. The Kolgorov-Smirnov test (KS test) was used to determine whether the distribution of the residuals was both approximately normal and met the assumption of the normality and linearity of the conditional distribution. Finally, it was confirmed that the Durbin-Watson values (DW) approached a value DW=2.00 in order to rule out autocorrelation problems in the errors [48,49].

All the tests were bilateral and were performed with a significance level of p<0.05. Data analysis was conducted with the SPSS-15 and Epidat 3.1 statistical software packages.

## Results

### Sample characteristics

There were 409 respondents, representing a response rate (RR) of 25.6%. RR were distributed as follows: 19.3% ‘TRS’, 36.5% ‘ASP’, 25.8% ‘GRH’ (p<0.001). The mean age of participants was 40.51 years (SD=9.09), with 44.4% males. The majority (78.1%) were in a stable relationship and 49.9% had children. 15.5% had achieved secondary or lower schooling, 52.1% had university degrees and 32.4% held doctorates. In terms of job position, 42.9% were ‘TRS’, 46.9% were ‘ASP’ and 10.2% were ‘GRH’. 40.6% of the participants worked ‘<35 h per week’, 26.8% worked ‘35-40 h’ and 32.6% worked ‘>40 h’. In terms of length of employment, 18.5% had been working at the university for ‘less than 4 years’, with 44.6% working ‘between 4 and 16 years’ and 36.9% for ‘more than 16 years’. The income distribution was as follows: 31.1% had a monthly income of ‘less than €1,200’, with 42.1% earning ‘€1,200-2,000’ and 26.8% ‘more than €2,000’. 67% of the participants had not taken sick leave in the previous year. 63.6% were permanent employees and the majority (93.8%) worked full time.

### Descriptive statistics, cronbach’s alpha and correlations

The BCSQ-36/BCSQ-12 subscales showed the following descriptive results: ‘involvement’ Md=4.92 (SD=0.84), ‘ambition’ Md=3.91 (SD=1.20), ‘overload’ Md=3.53 (SD=1.29), ‘indifference’ Md=2.58 (SD=1.20), ‘boredom’ Md=3.04 (SD=1.40), ‘lack of development’ Md=3.73 (SD=1.37), ‘lack of control’ Md=4.44 (SD=1.17), ‘lack of acknowledgement’ Md=4.42 (SD=1.42) and ‘neglect’ Md=2.52 (SD=0.90). The MBI-GS provided the following descriptive results: ‘exhaustion’ Md=2.39 (SD=1.42), ‘cynicism’ Md=2.07 (SD=1.59) and ‘efficacy’ Md=4.45 (SD=1.01). Table 2 shows the internal consistency obtained by the variables under study in this work, all with values α≥0.80. Table 2 also presents the r values for the raw or bivariate correlation between all the variables. As can be observed, all the BCSQ-36/BCSQ-12 dimensions showed significant associations with some of the standard dimensions of MBI-GS, most of which were moderately high.

**Table 2:** **Matrix of correlations and internal consistency for the BCSQ**-**36**, **BCSQ**-**12 and MBI**-**GS subscales**

	**1**	**2**	**3**	**4**	**5**	**6**	**7**	**8**	**9**	**10**	**11**	**12**
MBI												
1. Exhaustion	(0.92)											
2. Cynicism	0.63***	(0.92)										
3. Efficacy	−0.30***	−0.44***	(0.82)									
BCSQ												
4. *Overload*	0.57***	0.22***	−0.09	(0.86)								
5. Ambition	0.08	−0.08	0.26***	0.31***	(0.89)							
6. Involvement	−0.14**	−0.35***	0.45***	0.12*	0.34***	(0.80)						
7. *L*. *Development*	0.38***	0.60***	−0.22***	0.16**	<0.01	−0.24***	(0.88)					
8. Indifference	0.40***	0.65***	−0.49***	0.09	−0.18**	−0.50***	0.57***	(0.88)				
9. Boredom	0.25***	0.49***	−0.32***	0.03	−0.14**	−0.39***	0.64***	0.69***	(0.92)			
10. *Neglect*	0.32***	0.43***	−0.55***	0.10*	−0.20***	−0.65***	0.28***	0.66***	0.47***	(0.86)		
11. L. Acknowledgement	0.49***	0.59***	−0.23***	0.32***	<0.01	−0.16**	0.61***	0.40***	0.36***	0.25***	(0.88)	
12. L. Control	0.59***	0.53***	−0.29***	0.43***	0.09	−0.13*	0.41***	0.35***	0.21***	0.27***	0.57***	(0.81)

Variables 4–12 comprise the long version BCSQ-36. Variables 4, 7 and 10 (italics) comprise the short version BCSQ-12. α values in brackets in the diagonal. *** p<0.001; ** p<0.01; * p<0.05 (bilateral).

### Regression analysis

As seen in Tables 3, 4 and 5 the explanatory power of all models was reasonably high. The best explained dimension was ‘cynicism’, of which approximately 59% was captured by the BCSQ-36, and the worst explained was 'efficacy', of which 30% was captured by BCSQ-12. Compared to the BCSQ-12, the BCSQ-36 explained 9% more ‘exhaustion’ (Δ-adj-R^2^_y.123_=0.09; F=13.46; df_1_=6/df_2_=387; p<0.001), 15% more ‘cynicism’ (Δ-adj-R^2^_y.123_=0.15; F=24.53; df_1_=6/df_2_=387; p<0.001) and 7% more efficacy’ (Δ-adj-R^2^_y.123_=0.07; F=7.66; df_1_=6/df_2_=387; p<0.001). The fit of the multivariate linear regression models, evaluated by means of variance analysis, was statistically significant in all cases (p<0.001), with low standard error values (<1.20). DW values were all adequate (≈2.00), ruling out self-correction problems in errors. Residual distribution was approximately normal in all cases, except for the BCSQ-12 model in ‘cynicism’, which, nonetheless, showed a value that was very close to the criterion (p=0.048), making it generally possible to accept reasonably well the basic assumptions needed to go ahead with the regression legitimately.

**Table 3:** **Regression coefficients for the BCSQ**-**36 and BCSQ**-**12 models with regard to the** '**exhaustion**' **dimension of the MBI**-**GS**

**Model**/**variable**	**R**_**y**.**123**_	**R**^**2**^_**y**.**123**_	**adj**-**R**^**2**^_**y**.**123**_	**F** (**df**_**1**_/**df**_**2**_) **p**^**a**^	**Se**	**DW**	**p**^**b**^
BCSQ-36	0.74	0.54	0.53	51.01 (9/387) <0.001	0.98	1.82	0.604
	R_y3.12_	R_y(3.12)_	T	B (95% CI)	Se	Beta	p^c^
*Intercept*				−1.83 (−2.91 – -0.76)	0.55		0.001
Involvement	<0.01	<0.01	0.51	<0.01 (−0.16 – 0.16)	0.08	<0.01	0.971
Ambition	−0.04	−0.03	0.80	−0.03 (−0.12 – 0.06)	0.05	−0.03	0.475
Overload	0.44	0.34	0.70	0.44 (0.35 – 0.53)	0.05	0.40	<0.001
Indifference	0.18	0.12	0.34	0.25 (0.11 – 0.39)	0.07	0.21	<0.001
L. Development	0.04	0.03	0.39	0.04 (−0.07 – 0.15)	0.06	0.04	0.459
Boredom	−0.06	−0.04	0.41	−0.06 (−0.17 – 0.05)	0.06	−0.06	0.249
Neglect	0.05	0.03	0.40	0.09 (−0.08 – 0.26)	0.09	0.06	0.317
L. Acknowledgement	0.12	0.08	0.49	0.12 (0.02 – 0.21)	0.05	0.12	0.020
L. Control	0.29	0.20	0.57	0.33 (0.22 – 0.44)	0.06	0.27	<0.001
model/variable	R_y.123_	R^2^_y.123_	adj-R^2^_y.123_	F (df_1_/df_2_) p^a^	Se	DW	p^b^
BCSQ-12	0,67	0.45	0.44	105.96 (3/393) <0.001	1.06	1.87	0.177
	R_y3.12_	R_y(3.12)_	T	B (95% CI)	Se	Beta	p^c^
*Intercept*				−1.34 (−1.78 – -0.89)	0.23		<0.001
Overload	0.56	0.51	0.97	0.57 (0.48 – 0.65)	0.04	0.51	<0.001
L. Development	0.29	0.23	0.91	0.25 (0.17 – 0.33)	0.04	0.24	<0.001
Neglect	0.25	0.20	0.92	0.32 (0.20 – 0.44)	0.06	0.20	<0.001

R_y.123_=multiple correlation coefficient. R^2^_y.123_=coefficient of multiple determination. adj-R^2^_y.123_=adjusted coefficient of multiple determination. p^a^=p value for variance analysis associated with the regression. Se=standard error. DW=Dubin-Watson value. p^b^=p value for K-S test for normality contrast on residuals. R_y3.12_=partial correlation coefficient. R_y(3.12)_=semi-partial correlation coefficient. T=tolerance value. B=regression slope. CI=confidence interval. Beta=standardized slope. p^c^=p value of Wald test result. The sign < refers to absolute values.

**Table 4:** **Regression coefficients for the BCSQ**-**36 and BCSQ**-**12 models with regard to the** ‘**cynicism**’ **dimension of the MBI**-**GS**

**Model**/**variable**	**R**_**y**.**123**_	**R**^**2**^_**y**.**123**_	**adj**-**R**^**2**^_**y**.**123**_	**F** (**df**_**1**_/**df**_**2**_) **p**^**a**^	**Se**	**DW**	**p**^**b**^
BCSQ-36	0.77	0.60	0.59	64.43 (9/387) <0.001	1.02	2.04	0.211
	R_y3.12_	R_y(3.12)_	T	B (95% CI)	Se	Beta	p^c^
*Intercept*				−1.21 (−2.33 – -0.08)	0.57		0.036
Involvement	−0.10	−0.06	0.51	−0.16 (−0.33 – 0.01)	0.09	−0.08	0.059
Ambition	−0.03	−0.02	0.80	−0.03 (−0.12 – 0.07)	0.05	−0.02	0.560
Overload	0.03	0.02	0.70	0.03 (−0.07 – 0.12)	0.05	0.02	0.549
Indifference	0.34	0.23	0.34	0.53 (0.39 – 0.68)	0.07	0.40	<0.001
L. Development	0.17	0.11	0.39	0.20 (0.08 – 0.32)	0.06	0.17	0.001
Boredom	−0.03	−0.02	0.41	−0.04 (−0.15 – 0.08)	0.06	−0.03	0.501
Neglect	−0.04	−0.02	0.40	−0.07 (−0.24 – 0.11)	0.09	−0.04	0.465
L. Acknowledgement	0.24	0.16	0.49	0.25 (0.15 – 0.36)	0.05	0.22	<0.001
L. Control	0.22	0.14	0.57	0.26 (0.14 – 0.37)	0.06	0.19	<0.001
model/variable	R_y.123_	R^2^_y.123_	adj-R^2^_y.123_	F (df_1_/df_2_) p^a^	Se	DW	p^b^
BCSQ-12	0.67	0.45	0.44	106.12 (3/393) <0.001	1.19	2.00	0.048
	R_y3.12_	R_y(3.12)_	T	B (95% CI)	Se	Beta	p^c^
*Intercept*				−1.83 (−2.33 – -1.34)	0.25		<0.001
Overload	0.14	0.11	0.97	0.13 (0.04 – 0.22)	0.05	0.11	0.005
L. Development	0.55	0.48	0.91	0.59 (0.50 – 0.68)	0.05	0.51	<0.001
Neglect	0.34	0.27	0.92	0.49 (0.35 – 0.63)	0.07	0.28	<0.001

R_y.123_=multiple correlation coefficient. R^2^_y.123_=coefficient of multiple determination. adj-R^2^_y.123_=adjusted coefficient of multiple determination. p^a^=p value for variance analysis associated with the regression. Se=standard error. DW=Dubin-Watson value. p^b^=p value for K-S test for normality contrast on residuals. R_y3.12_=partial correlation coefficient. R_y(3.12)_=semi-partial correlation coefficient. T=tolerance value. B=regression slope. CI=confidence interval. Beta=standardized slope. p^c^=p value of Wald test result. The sign < refers to absolute values.

**Table 5:** **Regression coefficients for the BCSQ**-**36 and BCSQ**-**12 models with regard to the** ‘**efficacy**’ **dimension of the MBI**-**GS**

**Model**/**variable**	**R**_**y**.**123**_	**R**^**2**^_**y**.**123**_	**adj**-**R**^**2**^_**y**.**123**_	**F** (**df**_**1**_/**df**_**2**_) **p**^**a**^	**Se**	**DW**	**p**^**b**^
BCSQ-36	0.62	0.38	0.37	26.73 (9/387) <0.001	0.81	1.98	0.151
	R_y3.12_	R_y(3.12)_	T	B (95% CI)	Se	Beta	p^c^
*Intercept*				4.99 (4.11 – 5.88)	0.45		<0.001
Involvement	0.11	0.09	0.51	0.14 (0.01 – 0.28)	0.07	0.12	0.035
Ambition	0.18	0.14	0.80	0.14 (0.06 – 0.21)	0.04	0.16	<0.001
Overload	−0.06	−0.05	0.70	−0.05 (−0.12 – 0.03)	0.04	−0.06	0.232
Indifference	−0.15	−0.12	0.34	−0.17 (−0.29 – -0.06)	0.06	−0.20	0.003
L. Development	0.05	0.04	0.39	0.05 (−0.05 – 0.14)	0.05	0.06	0.346
Boredom	0.02	0.02	0.41	0.02 (−0.07 – 0.11)	0.05	0.03	0.690
Neglect	−0.23	−0.18	0.40	−0.33 (−0.47 – -0.19)	0.07	−0.29	<0.001
L. Acknowledgement	<−0.01	<−0.01	0.49	<−0.01 (−0.08 – 0.08)	0.04	<−0.01	0.974
L. Control	−0.14	−0.11	0.57	−0.13 (−0.22 – -0.04)	0.05	−0.15	0.006
model/variable	R_y.123_	R^2^_y.123_	adj-R^2^_y.123_	F (df_1_/df_2_) p^a^	Se	DW	p^b^
BCSQ-12	0.56	0.31	0.30	58.88 (3/393) <0.001	0.85	1.99	0.062
	R_y3.12_	R_y(3.12)_	T	B (95% CI)	Se	Beta	p^c^
*Intercept*				6.21 (5.86 – 6.56)	0.18		<0.001
Overload	−0.04	−0.03	0.97	−0.02 (−0.09 – 0.42)	0.03	−0.03	0.480
L. Development	−0.07	−0.06	0.91	−0.05 (−0.11 – 0.02)	0.03	−0.06	0.145
Neglect	−0.52	−0.51	0.92	−0.60 (−0.69 – -0.50)	0.05	−0.53	<0.001

R_y.123_=multiple correlation coefficient. R^2^_y.123_=coefficient of multiple determination. adj-R^2^_y.123_=adjusted coefficient of multiple determination. p^a^=p value for variance analysis associated with the regression. Se=standard error. DW=Dubin-Watson value. p^b^=p value for K-S test for normality contrast on residuals. R_y3.12_=partial correlation coefficient. R_y(3.12)_=semi-partial correlation coefficient. T=tolerance value. B=regression slope. CI=confidence interval. Beta=standardized slope. p^c^=p value of Wald test result. The sign < refers to absolute values.

Tables 3, 4 and 5 show the regression coefficients for all the models. As can be observed, the BCSQ-36 variables that contributed significantly to explaining ‘exhaustion’ were ‘overload’ (Beta=0.40; p<0.001), ‘lack of control’ (Beta=0.27; p<0.001), ‘indifference’ (Beta=0.21; p<0.001) and ‘lack of acknowledgement’ (Beta=0.12; p=0.020); those explaining ‘cynicism’ were ‘indifference’ (Beta=0.40; p<0.001), ‘lack of acknowledgement’ (Beta=0.22; p<0.001), ‘lack of control’ (Beta=0.19; p<0.001) and ‘lack of development’ (Beta=0.17; p=0.001); and those explaining ‘efficacy’ were ‘neglect’ (Beta=−0.29; p<0.001), ‘indifference’ (Beta=−0.20; p=0.003), ‘ambition’ (Beta=0.16; p<0.001), ‘lack of control’ (Beta=−0.15; p=0.006) and ‘involvement’ (Beta=0.12; p=0.035). The BCSQ-12 variables explaining ‘exhaustion’ were ‘overload’ (Beta=0.51; p<0.001), ‘lack of development’ (Beta=0.24; p<0.001), ‘neglect’ (Beta=0.20; p<0.001); those explaining ‘cynicism’ were ‘lack of development’ (Beta=0.51; p<0.001), ‘neglect’ (Beta=0.28; p<0.001), ‘overload’ (Beta=0.11; p<0.001); while ‘efficacy was only explained by ‘neglect’ (Beta=−0.53; p<0.001). The T values of variables were higher in the models developed from BCSQ-12 (>0.90) than in those from BCSQ-36 (0.34-0.80), meaning that they were models with less redundant variables for information purposes. Standard errors from slopes were low in all cases (<0.10). All intercepts were significant.

## Discussion

This study evaluated the explanatory power of an operationalized typological definition for burnout syndrome using the “Burnout Clinical Subtype Questionnaire”, in its long (BCSQ-36) and short (BCSQ-12) versions [19,22], regarding the standard offered by MBI-GS [6,47]. Multiple regression analysis enabled us to see that the dimensions of the MBI-GS were captured by the BCSQ-36 and BCSQ-12 subscales, with an adequate fit. Moreover, the distribution of residuals was approximately normal and no autocorrelation problems were detected.

As limitations, we should not overlook the fact that participant assessments were self-reported, and therefore may be weakened by socially desirable responses. Equally, the response rate obtained may seem low, although these values were similar to those found in other studies using similar on-line data collection procedures [45,46], and they enabled a sample size to be obtained that was not far off that initially estimated to be necessary, contributing evidence in relation to the aims originally set out. It should be pointed out that the distribution of the response rate was uneven for occupational strata, which could lessen the generalizability of our results. Finally, test-retest measurements were not taken for the variables under study, and therefore this aspect of their reliability could not be quantified. Nevertheless, we consider that the strength of this study lies in the work carried out with a broad and multi-occupational sample of employees in at-risk occupations with face-to-face personal contacts, in jobs with very different characteristics, which allows our conclusions to be generalized. Additionally, data quality was controlled by eliminating possible errors in the questionnaire transcription process through the use of purpose-designed software.

As we have explained previously, BCSQ-36 and BCSQ-12 were able to explain a high proportion of the variability contained in the criterion dimensions of the standard MBI-GS, although they were significantly higher in BCSQ-36, as we had established initially as a working hypothesis. All the dimensions of both typological models showed adequate internal consistency, and were significantly associated with some of the criterion dimensions of the standard on an individual basis. On the whole, the dimensions of the long and short typological models contributed to the explanation of each of the classic dimensions according to the proposed hypothesis, given that the “frenetic” profile presented the dimension that contributed most to the explanation of 'exhaution', as did the “underchallenged” profile with ‘cynicism’ and the “worn-out” profile with ‘efficacy’. However, as can be seen, the pattern of contributions obtained was somewhat more complex than initially expected.

First, ‘overload’ and ‘lack of control’ were the dimensions that basically explained ‘exhaustion’, something that is coherent with the Karasek’s demand–control model [50], according to which psychological strain is caused by the combination of high demands and low control. This result is also in line with the areas of worklife model [51], according to which workload and lack of control are important correlates of the syndrome, and with the more recent demands-resources model [52], in which personal resources are more important when coping with work-related demands. All of this is congruent with the process of stress caused by lack of control over results and over decision-making, with the association established between excess work and the appearance of fatigue and low levels of empathy, and with the development of emotional disorders caused by chronic stress [53-58]. We see that ‘lack of control’ contributed to the explanation of all the criterion dimensions, and that it can therefore be accepted as a key dimension when it comes to explaining the development of burnout symptoms in general, although it was in fact more strongly correlated with ‘exhaustion’.

On the other hand, ‘lack of development’ and ‘indifference’ were the dimensions that most contributed to explaining the criterion dimension of ‘cynicism’. Using Karasek’s framework with non-linear effects as proposed in a previous study [59], a manner of interpreting these results is that just as high demands may be overwhelming, or “toxic” to use Warr’s word [60], low demands may also be so unchallenging as to create feelings of frustration and monotony. This perspective is also included in the model by Schwab, Jackson and Schuler [37], which considers monotony to be an antecedent for the syndrome. Moreover, the ‘indifference’ variable contributed significantly to the explanation of all criterion dimensions, and therefore may be another key dimension for explaining the development of symndrome symptoms in general, although this variable was strongly correlated with ‘cynicism’ in particular, and both could eventually reduce satisfaction, interest and productivity in this subtype of workers [30,61-63].

Finally, ‘neglect’ and lack of ‘ambition’ were the dimensions that best explained the factor of lack of ‘efficacy’. These variables have also traditionally been associated with low performance levels in Bandura's theory of perceived self-efficacy and lack of it may also cause difficulties when it comes to alleviating perceived stress [43,64-66]. In general, it is understood that a progressive decrease in levels of engagement seems to be the kind of response adopted by burnout workers to cope with frustration, as described in the demand-resources model [32], and could be an important factor in explaining the differences between the subtypes from a longitudinal perspective [11-22]. These differences, explained by the BSCQ-36 and BCSQ-12 models by means of the degree of dedication to tasks as a criterion of typological classification, are not explained by previous models of burnout.

We have seen how that ‘overload’, ‘lack of development’ and ‘neglect’ variables of the BCSQ-12 contributed significantly to the explanation of ‘exhaustion’ and ‘cynicism’; however, of these three variables in BCSQ-36, only ‘overload’ contributed to that of ‘exhaustion’ and only ‘lack of development’ contributed to ‘cynicism’. This apparent inconsistency is the result of the control exerted by a number of variables over others when included together in the regression model. This effect can be understood if we observe that, while on a bivariate level significant correlations were obtained between the referred to independent and dependent variables (and generally between most of the variables under study), the ‘lack of development’ and ‘neglect' variables in the BCSQ-36 regression model did not provide new information on ‘exhaustion’ than that provided by the other variables. Likewise, no new information was provided by the ‘overload’ and ‘neglect’ variables on ‘cynicism’ in the BCSQ-36 regression model. This effect is clear if we observe the values provided by the partial and semi-partial correlation coefficients (R_y3.12_ and R_y(3.12)_). As previously mentioned, this is due to the information that could have been added in both cases being contained in the ‘indifference’, ‘lack of acknowledgement’ and ‘lack of control’ variables. We have already mentioned that ‘indifference’ and ‘lack of control’ could be dimensions with great explanatory power over all the classic symptoms, so they should perhaps be taken into account generally in the design of any intervention on the syndrome. However, ‘lack of acknowledgement’ was more important for explaining ‘exhaustion’ and ‘cynicism’ and not so much for lack of ‘efficacy’. These apparent inconsistencies did not occur in the models in relation to the ‘efficacy’ dimension, given that in both BCSQ-12 and BCSQ-36 the ‘overload’ and ‘lack of development’ variables did not contribute significantly to expaining it. In this case, 'neglect' was seen to be the dimension with the greatest explanatory power over lack of ‘efficacy’.

As we have seen, the explanatory power of BCSQ-36 was high and significantly greater than that of BCSQ-12. Given its length, complexity and the information it contributes, this questionnaire could be a very suitable instrument for use in mental health services, facilitating the design of interventions adapted to the characteristics of each particular case. For example, the “frenetic” subtype may benefit more from an intervention focusing on decreasing levels of activation, distress and fatigue. On the other hand, the “underchallenged” subtype may need to recover interest and enthusiasm to regain satisfaction and meaning with regard to the tasks assigned. Finally, the “worn-out” subtype needs to address feelings of hopelessness, lack of perceived efficacy and sense of abandonment at work. The source of the discomfort experienced in each subtype of burnout seems to come from very different coping strategies and dysfunctional attitudes based on the level of dedication at work [24]. In general, this approach is more in tune with how clinicians group symptoms and define disorders, something which may facilitate the use of specific forms of therapy. As Kokkinos [11] points out, the fact that each dimension of the syndrome is predicted by different variables should not remain unnoticed especially when designing and implementing intervention programmes to reduce burnout.

BCSQ-12 was also seen to have high explanatory power, very close to that of the long version. Given its brief and functional nature, and by making use of the already proposed cut-off points [27], it could be a very useful screening instrument in primary care consultations. In other words, this questionnaire could provide detection and recognition of burnout syndrome in cases where a commorbid association with anxiety, depressive or psychosomatic symptoms could lead to latent work-related psychosocial problems being overlooked [4]. We have seen that the subscales of ‘overload’, ‘lack of development’, and ‘neglect’ that comprise the BCSQ-12 were highly associated in a bivariate way with the criterion dimensions of ‘exhaustion’, ‘cynicism’, and ‘efficacy’ respectively, and contributed significantly to its explanation in multivariate models, while being relatively unrelated with each other [25], meaning that besides significant convergence, they present great discriminative power for differentiating the clinical subtypes. So, these subscales approach both burnout perspectives, that of typology and the traditional perspective. Taken separately, as they are presented in BCSQ-12, they could provide a brief description of the history of syndrome development in an operative way and with high convergent validity.

When these findings are seen within the context of accumulated clinical experience on burnout syndrome, it can be observed that as with other disorders (such as anxiety and depression), burnout appears to show itself in different ways, which require specific evaluation and possibly different intervention approaches [17-23]. Vercambre, Brosselin, Gilbert, Nerrière and Kovess-Masféty [67] take this perspective when they propose the use of different interventions depending on the characteristics presented by affected individuals. These authors recognize the multi-dimensional nature of burnout, but they set out their differential proposal over the classic dimensions of the MBI. These dimensions could include the core definition of burnout, but they do not facilitate a differentiation of the syndrome that would allow the history of the development of the disorder to be understood as is manifested in each particular case, something that can be done by means of the identification of the “frenetic”, “underchallenged” and “worn-out” subtypes of burnout. The properties making up the identified burnout subtypes may have different types of associations with the mediator variable of guilt, as suggested in other studies [26,68], thus contributing to explain the evolution of the different forms in which burnout is manifested [10], and perhaps enabling their influence on health to be differentiated [69]. Another interesting line of research that could lead to the establishment of specific biological markers for the syndrome may arise from the study of possible associations between the burnout subtypes and physiological correlates for the syndrome in current use, such as prolactin, cortisol, Immunoglobulin A, natural killer cell activity (NKCA) or mononuclear antibiodies CD16 and CD57 [70-73], which are associated with the functioning of the hypothalamo-pituitary-adrenal axis and the immune system.

## Conclusions

Both BCSQ-36 and BCSQ-12 present great explanatory power over the standard MBI-GS, with that of the former being significantly greater, which is understandable when taking into account the fact that it incorporates more information related to the antecedents of the classic or standard symptoms of burnout. In general, the BCSQ-36 may be very useful in mental health services, given that it provides a good deal of information, while the BCSQ-12 could be used as a screening measure in primary care consultations owing to its simplicity and functional nature. A definition of the development of burnout like that established using the BCSQ-36 and BCSQ-12 is a valid and useful tool for organizational evaluation and to identify work conditions to prevent the development of burnout and may provide a better understanding of the disorder as it is presented in each case, enabling the design of more specific treatment approaches. This perspective is more comprehensive than that provided by the classic MBI-GS, given that it assesses the individual’s perception of work conditions and enables a description to be made of the medical history of the development of the syndrome based on its particular idiosyncracy, providing a more complete characterization of burnout by means of clinical subtypes. The differences observed in the relative weighting of the properties of each of the burnout subtypes when it comes to explaining the standard dimensions suggest a pattern of contributions that may be of use for the development of new treatments when faced with the need for specific interventions. Results from interventions to deal with burnout have not been promising until now, although more research is required into the effectiveness of the programmes in use. More specific treatments based on a definition of the syndrome using clinical subtypes, based on the level of dedication to work-related tasks, could perhaps increase the efficacy of our interventions.

## Competing interests

The authors declare that they have no competing interests.

## Authors’ contributions

JMM, RA and JGC designed the project. JMM and BOB collected the data. JMM, PS and VMV performed the statistical analysis, and all authors interpreted the results, drafted the manuscript and read and approved the final manuscript.

## Pre-publication history

The pre-publication history for this paper can be accessed here:

http://www.biomedcentral.com/1471-2458/12/922/prepub
